# Keep a Little Fire Burning—The Delicate Balance of Targeting Sphingosine-1-Phosphate in Cancer Immunity

**DOI:** 10.3390/ijms23031289

**Published:** 2022-01-24

**Authors:** Catherine Olesch, Bernhard Brüne, Andreas Weigert

**Affiliations:** 1 Institute of Biochemistry I, Faculty of Medicine, Goethe-University Frankfurt, 60590 Frankfurt, Germany; catherine.olesch@dkfz-heidelberg.de (C.O.); b.bruene@biochem.uni-frankfurt.de (B.B.); 2 Bayer Joint Immunotherapeutics Laboratory, German Cancer Research Center (DKFZ), Im Neuenheimer Feld 280, 69120 Heidelberg, Germany; 3 Frankfurt Cancer Institute, Goethe-University Frankfurt, 60596 Frankfurt, Germany; 4 German Cancer Consortium (DKTK), Partner Site Frankfurt, 60596 Frankfurt, Germany; 5 Fraunhofer Institute for Translational Medicine and Pharmacology, Theodor-Stern-Kai 7, 60596 Frankfurt, Germany

**Keywords:** lipids, sphingosine-1-phosphate, inflammation, immunity, cancer

## Abstract

The sphingolipid sphingosine-1-phosphate (S1P) promotes tumor development through a variety of mechanisms including promoting proliferation, survival, and migration of cancer cells. Moreover, S1P emerged as an important regulator of tumor microenvironmental cell function by modulating, among other mechanisms, tumor angiogenesis. Therefore, S1P was proposed as a target for anti-tumor therapy. The clinical success of current cancer immunotherapy suggests that future anti-tumor therapy needs to consider its impact on the tumor-associated immune system. Hereby, S1P may have divergent effects. On the one hand, S1P gradients control leukocyte trafficking throughout the body, which is clinically exploited to suppress auto-immune reactions. On the other hand, S1P promotes pro-tumor activation of a diverse range of immune cells. In this review, we summarize the current literature describing the role of S1P in tumor-associated immunity, and we discuss strategies for how to target S1P for anti-tumor therapy without causing immune paralysis.

## 1. Introduction

### 1.1. Cancer

Cancer is a collective term for more than 100 diseases that are defined by the uncontrolled multiplication of cells and the invasion of these transformed cells into other parts of their tissue of origin, or into other tissues. Despite considerable progress in cancer detection, management, and therapy in the last decades, cancer remains the second leading cause of non-natural deaths worldwide [1]. Cancer is a genetic disease triggered by somatic mutations in cells, but the origins of these mutations are multifactorial with only about 5–10% being germline inherited genetic defects, while the remaining 90–95% arise due to environmental and lifestyle factors. Such factors are tobacco consumption, other air/environmental pollutants, diet, sun exposure, and infections [2,3]. While the contribution of some of these factors to overall mortality is in decline, the contribution of others, such as diet and its associated morbidities, e.g., obesity, are on the rise [4]. This indicates that the incidence of cancers related to the latter issues may increase in the coming years. Thus, new therapeutic approaches to fight cancer are needed.

### 1.2. The Tumor Microenvironment

Importantly, tumor initiation and development are multi-step processes that can require several decades in humans. They do not only depend on the tumor cells themselves, but they are shaped by the interaction of transformed cells with their local microenvironment. The tumor microenvironment (TME) is composed of cells such as vascular and lymphatic endothelial cells, pericytes, fibroblasts, and immune cells. An altered extracellular matrix and various gradients of nutrients and gaseous molecules, including oxygen, also contribute to the diversity of the TME [5]. The TME is decisively involved not only in tumor progression but also in therapy resistance [5]. Therefore, reshaping the TME to limit rather than support tumor growth provides promising opportunities for cancer therapy. A major component of the TME fitting this task is tumor-associated immune cells [3,6]. In clinically manifested tumors, these cells are often programmed by the TME to support tumor growth and metastasis through a multitude of mechanisms and signaling molecules, including bioactive lipids such as S1P. The virtue of re-educating the tumor-associated immune system to fight cancer has now been firmly established by the success of immune checkpoint blockade (ICB) [7,8,9,10]. In this review, we summarize the current understanding how the immune system affects tumor development and point out directions of how interfering with S1P production and signaling might be instrumental in promoting anti-tumor immunity.

## 2. The Immune System in Cancer

### 2.1. Tumor-Promoting Inflammation

It is now firmly established that the immune system shapes tumor development at each stage [11]. However, different qualities of tumor-associated immune responses can either result in rejection or progression of tumors. Generally, the physiological microenvironment of any given organ is designed to be tumor-suppressive. This property can be subverted by chronic inflammation that arises as a consequence of the environmental and lifestyle factors introduced above, thus favoring malignant transformation [12,13]. For instance, inflammation was recently shown to elicit a memory response in pancreatic epithelial cells, allowing them to adapt to future inflammatory events. This response was solidified by somatic *KRAS* mutations. On the downside, epithelial memory together with *KRAS* mutations are not only protected from future inflammation-related injury but are also predisposed to the development of cancer [14]. Somatic mutations themselves can also arise as a consequence of chronic inflammation among others by activating innate immune cells to produce genotoxic agents such as oxygen or nitrogen radicals [15,16,17]. Accordingly, anti-infectious and anti-inflammatory drugs have been linked to a reduced risk to develop certain, although not all, types of cancer [18].

### 2.2. Anti-Tumor Immunity

Once cancerous growth is initiated, altered self-patterns, including neoepitopes and stress-related cell surface architecture, can be recognized by the anti-tumor arm of the immune system. This process presumably occurs regularly in humans, leading to eradication of initial cancerous lesions or at least immune control (immune equilibrium). The theory of cancer immunoediting predicts that if eradication fails, the constant interaction between immunity and the malignant cells during the equilibrium phase may result in the development of a highly immunosuppressive tumor phenotype that allows immune escape and, consequently, tumor growth [19,20,21,22,23]. This notion is supported by recent data showing that the adaptive immune system may be involved in shaping the mutational landscape in human tumors, as suggested by mutagenesis studies in mice with and without a functional adaptive immune system [24]. Thus, mutations in clinically detectable tumors, particularly in tumor suppressor genes, already reflect the tumor’s need to avoid the restrictive influence of the adaptive immune system, which is also indicated by studies showing ‘epigenetic hiding’ of neoepitopes in lung cancer [25]. Evidence of active anti-tumor immunity was long debated but is now unchallenged, at least in most tumor entities. Bioinformatic analyses revealed that the immune contexture in tumors has powerful prognostic and predictive potential in cancer, both at primary and secondary sites [26,27]. Moreover, immune evasion signatures precede tumor invasion in lung cancer [28], which supports the theory of cancer immune editing. The cytotoxic potential of lymphocytes such as natural killer (NK) cells is associated with cancer risk [29]. Transplant patients receiving immunosuppressive treatment show an increased incidence of cancer [30] that may even stem from the donor organ years after the donor was supposedly cured of cancer [31]. Moreover, neoantigens can trigger immune reactions to the native protein in human cancer patients resulting in autoimmunity [22]. Last but not least, re-activation of anti-tumor immunity by ICB has shown remarkable clinical efficacy in cancer patients [8,32]. Immune checkpoints regulate the continuation versus termination of adaptive immune responses. Immune checkpoint inhibition targets negative immune checkpoints by using neutralizing antibodies that disrupt, e.g., the interaction of programmed cell death 1 (PD-1) on lymphocytes with programmed death-ligand 1 (PD-L1). Both proteins are upregulated as negative feedback following lymphocyte activation to terminate inflammation. Immune checkpoint inhibition, thus, reactivates anti-tumor immunity [32]. Interestingly, lifestyle choices associated with chronic inflammation may also actively suppress baseline and therapy-induced anti-tumor immunity. Diet was recently shown to not only affect tumor-promoting inflammation, but also to determine the response to ICB by modulating the intestinal microbiome, which likely feeds back into altered inflammation in response to microbial-derived pathogen-associated molecular patterns [33]. Obesity, a potential consequence of a western diet that causes chronic inflammation linked to cancer [34,35], can also suppress the anti-tumor immune response by causing metabolic adaptations in the TME [36]. Thus, avoiding lifestyle and environmental factors that promote tumorigenesis may also aid in preventing tumor immune escape and improving anti-tumor therapy.

### 2.3. The Tumor-Supporting Microenvironment

While the immune system is actively engaged in protection against transformed cells in humans, this protection obviously has failed in clinically detectable tumors. Loss of immune protection may involve shaping the mutational landscape [24,25], but the immunosuppressive nature of the microenvironment in a growing tumor is also driven by factors other than its acquired somatic mutations. Numerous processes such as adaption to the metabolic situation in a tumor including lack of nutrients and hypoxia, the interaction with dying tumor cells, and negative feedback signals that are initiated after induction of immune responses and that normally serve to limit autoimmunity during infection, result in educating resident immune cells to actively support tumor growth [12,37,38]. Consequently, tumors contain a mixture of tumor-promoting and tumor-suppressive immune cell populations, and the former increase in impact during tumor immuno-editing [28]. Density and tumoricidal activity of cytotoxic lymphocytes such as γδ T cells, CD8+ T cells, T helper 1 (TH1)-polarized CD4+ T cells, memory T cells or NK cells, as well as activated myeloid cells are associated with a favorable prognosis. In contrast, the presence of suppressive myeloid cells such as macrophages or myeloid-derived suppressor cells, and lymphocytes such as regulatory T cells (Treg) or TH17-polarized CD4^+^ T cells are often linked to poor prognosis [39,40,41]. This dichotomy of a cell subset being polarized to an either pro- or anti-tumor phenotype is true for most immune cell populations found in tumors. For instance, macrophages with a polarized pro-inflammatory phenotype are often connected with favorable prognosis, while macrophages with a polarized anti-inflammatory phenotype are connected with poor prognosis [40,42]. A similar picture emerges for tumor-associated neutrophils and dendritic cells (DCs) [43,44,45]. Based on this strong connection of the phenotype of the tumor-associated immune system with patient survival and the clinical success of ICB, one may propose that tumor therapy in general should aim at tipping the immune balance towards anti-tumor properties, or at least try to avoid limiting anti-tumor immunity. Unfortunately, cytotoxic therapy targeting rapidly dividing cells often induces lymphopenia [46]. This is clearly undesired because the peripheral immune system may not only be required for baseline anti-tumor immunity but for protective immunity after ICB as well [47,48]. In the following paragraphs, we summarize the potential of targeting the S1P axis in cancer with a focus on this very question. Can we maintain or even exploit immune cell function in this process? To be able to discuss this question, we explored Pubmed using the following search terms: sphingosine-1-phosphate, S1P, S1PR1/2/3/4/5, sphingosine kinase, SPHK1/2, S1P Lyase, SGPL1, S1P phosphatase, SGPP1/2, PLPP3 in combination with cancer and immunity, immune, or inflammation in all possible permutations. We used the same search terms to receive information on clinical trials related to S1P and cancer at https://clinicaltrials.gov, accessed on 19 January 2022. From the resulting studies, we prioritized those that directly addressed the role of S1P in tumor immunity and inflammation. Due to this strategy, publications describing the role of S1P metabolism and signaling in cancer unrelated to cancer-associated immunity or inflammation are underrepresented. Another limitation of our approach is that studies exploring S1P metabolism and signaling in an immune context unrelated to cancer are not prominently discussed. Thus, potentially interesting hypotheses on how S1P affects tumor immunity based on extrapolation from other immune contexts are largely absent in the text below.

## 3. S1P Signaling and Immune Cell Dynamics

### 3.1. S1P Metabolism and Signaling

**Figure 1:**
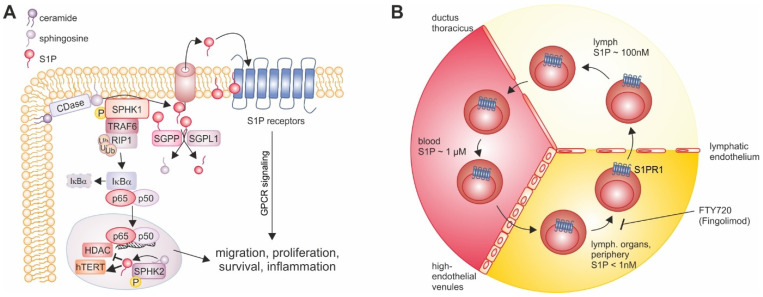
S1P metabolism, signaling, and role in immune cell trafficking. (**A**) S1P is produced at biological membranes from sphingosine-by-sphingosine kinases. Intracellularly, it acts as a co-factor for inflammatory signaling via NF-κB (p50/p65) for inhibiting HDACs and promoting hTERT activity. Intracellular S1P is rapidly degraded or released from cells to couple to specific G-protein coupled receptors. (**B**) Lymphocytes in lymphatic organs express S1PR1, allowing them to follow the S1P gradient towards lymph, from where they enter the blood stream. High S1P levels trigger internalization of S1PR1, allowing recirculation into lymphatic organs, where S1PR1 is exposed at the cell surface again. Downregulating cell surface S1PR1 with drugs such as FTY720 traps lymphocytes in lymph nodes. Details can be found in the main text. Abbreviations: CDase, ceramidase; HDAC, histone deacetylase; hTERT, telomerase reverse transcriptase; IκBα, nuclear factor of kappa light polypeptide gene enhancer in B-cells inhibitor, alpha; RIP, Receptor-interacting serine/threonine-protein kinase; S1P, sphingosine-1-phosphate; S1PR, S1P receptor; SGPL, S1P lyase; SGPP, S1P phosphatase; SPHK, sphingosine kinase; TRAF, TNF receptor associated factor.

The sphingolipid S1P is a bioactive signaling molecule with powerful impact in physiological as well as pathophysiological settings, which is why its levels are tightly regulated by a number of mechanisms [49,50,51,52,53,54] (Figure 1A). Sphingolipid metabolism starts with the generation of ceramide via condensation of serine and palmitoyl-CoA to form 3-keto-dihydrosphingosine, which is subsequently reduced to dihydrosphingosine and N-acylated to form the large group of dihydroceramides [55,56]. Ceramide desaturase then converts dihydroceramides to ceramides. Ceramides as the primary building blocks of sphingolipid anabolism are then either phosphorylated to generate the signaling molecule ceramide-1-phosphate, glycosylated to form glucosylceramides, or converted to sphingomyelin, the latter two lipid classes being integral parts of the outer leaflet of the plasma membrane. Sphingomyelin processing by sphingomyelinases creates ceramide in a reverse reaction, which can be further degraded by ceramidases to generate sphingosine. Sphingosine in turn can be phosphorylated by sphingosine kinase-1 (SPHK1) or -2 (SPHK2) to form S1P [52,57].

These enzymes differ in subcellular localization, with SPHK1 being predominantly found in the cytosol and being able to translocate to the plasma membrane. SPHK2 is found at a number of intracellular membranes, such as the endoplasmic reticulum and the mitochondria, and is able to shuttle into and out of the nucleus [58]. S1P, which was first recognized as an intermediary product of terminal sphingolipid catabolism, can be degraded by S1P lyase (SGPL1) to hexadecenal and phosphoethanolamine [59]. Alternatively, S1P can be dephosphorylated by S1P phosphohydrolase 1 and 2 (SGPP1/2), or the non-specific lipid phosphatase lipid phosphate phosphatase 3 (PLPP3), to enter the salvage pathway back to sphingosine and ceramide [59,60]. S1P degradation or dephosphorylation keep intracellular S1P at low levels in most tissues under physiological conditions [59], which is disturbed under pathological conditions such as during inflammation and in cancer. In this context, the so far identified intracellular actions of S1P may become relevant. During inflammation, S1P is required for signaling via TNF-α receptor-associated factor 2 (TRAF2), an E3 ubiquitin ligase of the nuclear factor ‘kappa-light-chain-enhancer’ of activated B-cells (NF-κB) pathway [61], and is a co-factor for the inhibitor of apoptosis 2 (cIAP2), which promotes polyubiquitination of interferon regulatory factor-1 to enhance chemokine expression [62], while ceramide synthase 2 is inhibited by S1P [63] to regulate inflammation [64,65]. In cancer cells, S1P produced by SPHK2 acts as an inhibitor of the class I histone deacetylases HDAC1 and HDAC2 to enhance gene transcription [66], particularly genes activated by hypoxia [67], and couples to the catalytic subunit of telomerase to enable tumor cell replication and avoid senescence [68]. Moreover, S1P produced by both SPHK1 and 2 activates an atypical protein kinase C to improve cell survival [69].

Besides its intracellular actions, S1P can be exported from cells to serve as a ligand for five G-protein coupled receptors (S1PR1-5), triggering autocrine or paracrine signaling [70]. S1P transporters include certain ABC family transporters and spinster 2 (SPNS2) in a number of cells [71,72,73,74], while the major facilitator superfamily transporter 2b (MFSD2B) specifically mediates S1P export from red blood cells and platelets [75]. Major sources of S1P under physiological conditions are red blood cells and endothelial cells resulting in relevant concentrations of S1P only in blood (~1 µM) and lymph (~100 nM), where the majority is bound by chaperones such as albumin or HDL to enhance solubility of the lipid [59,76,77]. The concentration gradient between high S1P levels in the circulation and its virtual absence in other tissues is key to its main physiological functions, which are immune cell trafficking and regulating vascular tone and integrity [78,79]. S1PR1-5, as it is often observed for families of G-protein coupled receptors sharing a ligand, display cell-type-specific expression patterns. While S1PR1, 2, and 3 are expressed ubiquitously, S1PR4 and 5 show tissue-specific distribution. S1PR4 is predominantly found in hematopoietic tissues and endothelial cells under basal conditions [80,81], whereas S1PR5 expression is restricted to NK cells [82], DCs [83], the central nervous system [84], endothelial cells [85], and certain cancer cells [86,87], indicating specialized functions of these two S1PRs. Moreover, S1PRs are linked to distinct G proteins [78]. Thus, it is not surprising that signaling by distinct S1PRs can mediate opposing functional responses [78]. Along this line, S1PR1 promotes lymphocyte migration and stabilizes the endothelial barrier, while S1PR2 counteracts these effects [88,89]. However, there is also redundancy in the system. This notion and the importance of extracellular S1P signaling per se is revealed by observations that both SPHK1 and 2, as well as S1PR1/2 and 3, are required for vascular development during embryogenesis [90,91,92].

### 3.2. S1P and Immune Cell Trafficking

A major physiological role of S1P, the regulation of immune cell trafficking [93] (Figure 1B), was revealed by the observation that the immunosuppressive agent FTY720 exerts its action by disrupting the ability of T cells to follow the S1P gradient towards the circulation. This resulted in trapping them in primary and secondary lymphatic organs [94,95]. S1PR1 expression on T cells is induced during thymic development, enabling their egress into the bloodstream [96]. There, S1P coupling to S1PR1 triggers reversible receptor endocytosis. This allows T cells to follow other chemotactic signals into peripheral tissues, where the lack of S1P results in S1PR1 re-localization to the plasma membrane. Cell surface S1PR1 allows T cells to migrate back into the circulation via the lymphatics, from where they enter the bloodstream, which completes the cycle. This elegant system ensures lymphocyte surveillance of all tissues. Among S1P transporters, SPNS2 plays a major role in lymphocyte trafficking. *SPNS2*-deficient mice displayed reduced circulating S1P levels coupled with lymphopenia [97]. As a consequence, a number of auto-inflammatory conditions including delayed-type contact hypersensibility, dextran sulfate sodium (DSS)-induced colitis, experimental autoimmune encephalopathy and collagen-induced arthritis were suppressed upon SPNS ablation, which was attributed to reduced levels of effector lymphocytes in the affected organs [97]. The importance of the S1P/S1PR1 axis in T cell homeostasis is further supported by the observation that tissue residency of memory T cells requires permanent downregulation of S1PR1 [98]. This occurs via the induction of the lymphocyte activation marker CD69, which induces S1PR1 internalization [98,99]. S1PR1 internalization also occurs during effector T cell activation, where it serves the same purpose of retaining these cells in their current environment to enable them to complete their specific tasks [100]. This larger pattern of S1PR-dependent lymphocyte trafficking is mirrored by most immune cell subsets, although sometimes in a more complex manner or by using alternative S1P receptors. NK cell migration towards S1P occurs via S1PR5 rather than S1PR1 [82], while monocytes respond to S1P via S1PR3 [101] or S1PR5 [102]. B cell positioning in different zones of secondary lymphoid organs depends on local S1P gradients that are created by lymphatic endothelial cells and sensed via S1PR1-3 [96,103]. How granulocytes respond to S1P gradients is still not entirely resolved [104]. Mature myeloid cells show different S1P receptor expression levels dependent on their activation. Immature DCs express S1PR2 and 4, while upon maturation, S1PR1 and 3 are predominantly expressed. In this manner, mature DCs escape the inhibitory action of S1PR2, which allows them to emigrate into lymphatic tissues to present antigens to lymphocytes [105]. In macrophages, S1PR2 is expressed during inflammation with S1PR1 being upregulated during its resolution to facilitate their emigration from the site of inflammation [106,107]. These examples mirror the situation in T cells and allow the conclusion that S1PR1 levels determine permanent or transient tissue residency of immune cells. Modulating the functional S1P gradient by FTY720 (Fingolimod) is clinically used to treat patients with relapse-remitting multiple sclerosis and the principle of modulating S1P receptor signaling to avoid lymphocytes reaching sites of auto-inflammatory tissue destruction, e.g., in inflammatory bowel disease, inflammatory skin diseases, is extensively explored in clinical trials [79].

The S1P/S1PR axis that maintains lymphocyte trafficking can be disrupted during inflammation, as indicated above for T cells. This occurs not only through regulating S1PR expression but also via an increase in S1P production [54]. When produced in such an inflammatory context, S1P affects immune cell parameters other than migration, including cell survival, differentiation, and activation. These features will be discussed below in the context of cancer and have been summarized before [53,60,78].

## 4. S1P Signaling and Tumor Immunity

### 4.1. S1P Promotes Cancer Development

As indicated above, S1P signaling regulates the migration, survival, and proliferation of cells. S1P levels in tumors are often elevated either due to increased expression of SPHKs, inflammatory signaling in the TME, tumor enriched environmental cues such as hypoxia, or tumor cell death [108,109,110,111,112]. Moreover, S1P regulates vascular development, which suggested a potential role for tumor vascularization. In turn, leaky blood vessels resulting from dysregulated angiogenesis, which is a characteristic of tumor angiogenesis, are another potential source of S1P in tumors [113]. Thus, it appeared natural to consider S1P as a pharmacological target in cancer [108]. To date, a number of early clinical trials have been performed to that end. The SPHK1 inhibitor Safingol in combination with cisplatin was tested in a phase 1 study in patients with locally advanced or metastatic solid tumors [114]. The data of this trial were published in 2011, but since then phase 2 trials with Safingol have not been initiated. In a phase 1 trial, the SPHK2 inhibitor ABC294640 (Opaganib) showed promise for treating patients with cholangiocarcinoma [115]. Consequently, a phase 2 clinical trial (NCT03377179) for treating patients suffering from advanced cholangiocarcinoma with Opaganib alone and in combination with hydroxychloroquine sulfate has been started, which has recently been expanded to include previously excluded patients (NCT03414489). Moreover, a phase 2 trial with Opaganib as an additive to androgen antagonists in metastatic castration resistant prostate cancer patients is under way (NCT04207255). The S1PR modulator Fingolimod has been tested in combination with radiation and Temozolomide in a phase 1 study in newly diagnosed high grade glioma patients (NCT02490930). The results of this trial are not publicly available. Finally, the monoclonal anti-S1P antibody sonepcizumab was administered to patients with advanced solid tumors in a phase 1 trial (NCT00661414). After promising results, a phase 2 study in patients with metastatic renal cell carcinoma was conducted [116]. Despite not meeting its primary endpoint concerning progression-free survival, this study suggested an improved overall survival. Preclinical data with sonepcizumab had shown reduced cancer cell survival and diminished tumor angiogenesis in transplanted tumor models [117]. While a survival-promoting role of extracellular S1P is largely undoubted, the mechanisms underlying reduced blood vessel infiltration into these transplanted tumors remains obscure. Interrupting a direct impact of S1P on endothelial cells appears unlikely, since S1P was recently shown to stabilize tumor-associated blood vessels via endothelial S1PR1 (as well as S1PR2/3) signaling. Augmented tumor development and metastasis associated with deficient vessel maturation was observed in mice lacking *S1PR1* in endothelial cells, while overexpression of *S1PR1* in endothelial cells normalized tumor vessels and improved the response to tumor therapy [118]. Other S1P targets affecting angiogenesis may include the tumor cells themselves or microenvironmental cells including immune cells [107,119]. However, these controversial effects of S1P on the tumor-associated vasculature may be one reason why systemic targeting of S1P in clinical trials has not produced the expected success. Another reason might be that targeting systemic S1P with sonepcizumab was shown to induce peripheral lymphopenia [116], which may have led to suppressing anti-tumor immunity. How this may be avoided will be discussed systematically in the following paragraphs, where we summarize the contribution of individual components of the S1P production and signaling machinery to tumor-associated immune responses.

### 4.2. Sphingosine Kinases and Tumor Immunity

As opposed to global scavenging of S1P, inhibition or deletion of either of the two sphingosine kinases is not expected to result in lymphopenia due to the compensatory effect of the remaining kinase. Indeed, both kinases need to be targeted to reach meaningful changes in plasma S1P levels [92,120]. Therefore, adverse effects on anti-tumor immunity by individual sphingosine kinase inhibition have not been reported, but both enzymes appear to be involved in tumor-promoting inflammation. *SPHK1* expression was increased in human colon cancer samples compared to normal colon mucosa, which was further increased in metastatic cancer. Moreover, azoxymethane (AOM)-driven colon cancer in mice increased blood levels of S1P, and *SPHK1*-deficient mice had reduced colon cancer development [121]. Increased S1P levels induced by compensatory upregulation of *SPHK1* upon *SPHK2* ablation in mice were also observed in the AOM/DSS model of colitis-associated cancer (CAC). S1P triggered activation of resident myeloid cells via an NF-κB/IL-6/signal transducer and activator and transcription 3 (STAT3) loop that involved S1PR1 [122]. Indeed, elevated SPHK1 levels were also observed in human CAC patients compared to patients with sporadic colorectal cancer (CRC), although no correlation with activated STAT3 or IL-6 was found [123]. Interestingly, also SPHK2 may be involved in CAC, since the SPHK2 inhibitor ABC294640 prevented the development of AOM/DSS tumors and altered the phosphatidylinositol 3-kinase (PI3K)/protein kinase B (AKT) pathway not only in epithelial but also infiltrating inflammatory cells [124]. Interestingly, PI3Kγ was identified as a myeloid immune checkpoint molecule whose targeting reduced immunosuppressive and pro-tumorigenic myeloid cell properties [125]. This fits to observations in breast cancer xenografts where ablation of *SPHK2* in tumor cells reduced pro-tumor features of infiltrating macrophages [126]. SPHK1 was connected to breast cancer-associated immune responses as well. A study investigating human breast cancer found elevated S1P levels in tumors compared to peritumoral or normal human breast samples to correlate with elevated *SPHK1* gene expression. In turn, expression of *SPHK1* was associated with an increased expression of immune-related genes such as *CD68*, *CD163*, *CD4*, and forkhead box P3 (*FOXP3*), indicating increased infiltration of macrophages and Treg [127]. Both immune cell populations can suppress effector T cell responses [128,129]. In melanoma, SPHK1 was also connected to Treg abundance. Expression of *SPHK1* in melanoma cells was associated with shorter survival in metastatic melanoma patients treated with anti-PD-1 ICB. SPHK1 targeting augmented the response to ICB in murine models of melanoma, breast, and colon cancer and limited Treg infiltration [130]. Moreover, ablation of *SPHK1* in melanoma cells lead to a shift of macrophages with tumor-promoting to macrophages with tumor-suppressive phenotype markers, which was coupled to T cell recruitment and activation [131]. Unconventional T cells were also activated upon *SPHK1* ablation in Mantle cell lymphoma cells. An induction of natural killer T (NKT) cell activation was observed under these conditions accompanied by an increase in the NKT cell lipid antigen cardiolipin upon *SPHK1* ablation [132]. Together, these studies suggest that targeting any SPHKs may remove immunosuppressive features in the TME. However, since both kinases may promote cell proliferation, the question remains whether targeting SPHKs may interfere with adaptive immune cell expansion, which is required for adequate adaptive immune responses. There is currently no direct evidence supporting this assumption. Rather, *SPHK1*-deficient T cells showed a sustained memory response and reduced differentiation to Tregs independent of S1PR signaling, which resulted in activity of T cells against murine melanoma cells and synergy with ICB [133]. In conclusion, targeting SPHKs, particularly SPHK1 in combination with ICB, may be of interest to combine inhibition of cancer cell expansion with stimulation of anti-tumor immunity.

### 4.3. S1P Degrading Enzymes and Tumor Immunity

Given the evidence that increasing S1P by SPHKs may limit anti-tumor immune responses and support tumor-promoting inflammation, one may assume that preventing S1P degradation has similar effects. So far, data supporting this notion have emerged mainly from studies where *SGPL1* was genetically ablated in mouse CAC models. Mice lacking *SGPL1* in intestinal epithelial cells showed enhanced S1P levels and tumor growth, accompanied by increased STAT3 activation and inflammatory cytokine levels, which were inhibited when colonic *SGPL1* levels were increased in WT mice [134]. Interestingly, ablation of *SGPL1* in myeloid cells also increased S1P levels. Hereby, immune cell specific *SGPL1* ablation caused massive immune cell infiltration, delayed tumor formation, and a mix between the previously observed STAT3 pattern and immunosuppressive marker expression. In contrast, *SGPL1* ablation in non-myeloid cells elicited rapid formation of colon tumors accompanied by a tumor-favoring microenvironment. Thus, the cellular source of S1P is decisive for triggering inflammation-induced cancer or cancer-induced inflammation, respectively [135]. In contrast to SGPL1, data on S1P phosphatases in experimental tumor models are lacking. So far, bioinformatics analyses revealed a positive association of *SGPP1* and *PLPP3* expression with relapse-free survival in triple-negative breast cancer patients. Expression of both enzymes correlated with tumor-infiltrating DCs, CD4+ and CD8+ T cells, neutrophils, and macrophages [136]. Moreover, expression of *SGPP1* and *PLPP3* was associated with overall survival in lung adenocarcinoma and non-small-cell lung carcinoma (NSCLC) patients, where expression of *PLPP3* correlated with tumor-infiltrating immune cells in NSCLC patients [137]. Such patterns of expression correlation can also reflect an altered cellular composition when the enzymes are differentially expressed in cell types and subtypes. Thus, functional studies are required to test the role of S1P phosphatases in tumor immunity.

### 4.4. S1P Export and Tumor Immunity

Besides production and degradation, S1P export decisively contributes to its signaling properties. Data from different human hepatocellular carcinoma (HCC) cohorts showed that both *SPHK1* and the S1P exporter *ABCC1* were expressed at higher levels in aggressive HCC when compared with normal liver or cirrhotic tissue. High expression of these genes, which may indicate S1P export, correlated among others with immune signatures related to TNFα and IL6 signaling but also to allograft rejection and IFN-γ, which has anti-tumor properties. Moreover, an association with mixed anti- and pro-tumor immune cell infiltrates was found. Nevertheless, high virtual S1P export was associated with worse disease-specific survival and overall survival [138]. Again, functional studies are needed to clarify the role of ABCC1-dependent S1P release in anti-tumor immunity. In contrast, recent data indicate an unexpected role for SPNS2 in metastasis and tumor-associated immunity. *SPNS2*-deficient mice exhibit peripheral lymphopenia and defects in B cell homing to secondary lymphoid organs, which results in impaired humoral immunity upon immunization with E. coli and suggests SPNS2 as a target for immunosuppressive therapy [139]. Despite these findings, global and lymphatic endothelial *SPNS2* ablation reduced the formation of experimental metastases in mice, while increasing NK cells and CD8+ T cells at the metastatic sites [140]. These results were reproduced with an inhibitor of SGPL1 [140]. In an iron-deficient mouse model of HCC, overexpression of SPNS2 also increased HCC metastasis, which was reduced upon *SPNS2* ablation. However, these effects were immune cell independent, even though depleting *SPNS2* again increased NK cells and effector T cells in the lungs [141]. These local immunostimulatory effects of *SPNS2* ablation, despite its overall immunosuppressive effect during immunization, warrant further investigation. Interestingly, S1P independent features of SPNS2 have been proposed as well. *SPNS2* expression was upregulated in colon adenoma and CRC compared to normal tissues. However, low *SPNS2* expression correlated with poor prognosis in CRC and ectopic expression of *SPNS2* inhibited cell proliferation, migration, epithelial-mesenchymal transition, invasion, and metastasis in CRC cell lines. This appeared to be S1P independent, rather acting directly via inactivation of AKT signaling [142]. These data also need to be considered when proposing SPNS2 as a target to improve anti-tumor immunity and combat metastasis.

### 4.5. S1P Receptors in Tumor Immunity

Given the above-mentioned, sometimes antithetic properties of S1P signaling through its individual receptors, targeting S1P metabolizing enzymes, may be expected to result in reduced efficacy compared to targeting S1PRs selectively. Thus, S1PR-specific effects on anti-tumor immunity need to be elucidated.

#### 4.5.1. S1PR1

S1PR1 was the first S1PR to be discovered and is certainly the best explored receptor through which S1P exerts its immunomodulatory functions. Its importance in regulating T cell trafficking via the circulation [96] would lead to the expectation of reduced anti-tumor immunity once S1PR1 is targeted. However, its role in tissue-resident memory T cells (Trm), which are found in tumors and require long-lasting S1PR1 internalization raises doubts concerning this hypothesis. Absence of S1PR1 is well-established as a marker for identification of Trm in tumors. Trm abundance positively correlates with responsiveness to ICB and therapy success in cancer patients [143,144]. For another T cell subset, namely Tregs, S1PR1 expression is also an important cue for migration to the tumor site. Data from a syngeneic tumor model with CD4+ T cell-specific ablation of *S1PR1* indicate that S1PR1 on CD4+ T cells regulates intratumoral Treg expansion leading to CD8+ T cell suppression and tumor progression through STAT3-dependent activation of Tregs [145]. The role of S1PR1 in the accumulation of tumor-specific Tregs was underpinned in studies with breast cancer patients, demonstrating that S1PR1-dependent decreased Treg levels in the bone marrow correlated with increased tumor antigen-specific Treg redistribution to the tumor. Mechanistically, bone marrow-resident antigen presenting cells in addition to T cell receptor stimulation mediated S1PR1 upregulation on Tregs [146]. Consequently, one might argue that it may be beneficial for cancer patients to target S1PR1 in order to prevent enhanced accumulation of pro-tumor Tregs or enhance Trm abundance within tumors. However, in other tumor entities such as glioblastoma and other intracranial tumors, S1PR1 downregulation is an important tumor-mediated mechanism of T cell dysfunction by trapping T cells in the bone marrow and preventing their migration to the tumor site [147]. Besides T cells, S1PR1 was also involved in STAT3 activation in tumor-associated myeloid cells [148]. Reduction of S1PR1-STAT3 signaling diminished pro-tumor cytokine production, including IL-6, which resulted in reduced tumor progression in mouse models of bladder cancer and CAC [134,149]. Furthermore, S1PR1-STAT3 signaling in myeloid cells was important for the establishment of a pre-metastatic niche to pave the way for the effective colonization of tumor cells and tumor outgrowth at distant sites [150]. This was also observed in a model of obesity-dependent breast cancer, where obesity was sufficient to increase S1P. S1P signaled through S1PR1 to promote pro-inflammatory cytokine expression and macrophage infiltration, favoring tumor progression and the induction of the premetastatic niche in lungs [151]. However, S1PR1 also regulated macrophage-dependent metastasis formation independently of STAT3 signaling. Macrophage-specific S1PR1 signaling promoted NLR family pyrin domain containing 3 (*NLRP3*) expression leading to enhanced IL-1β production via the inflammasome, which resulted in enhanced lymphangiogenesis and tumor metastasis in a murine breast cancer model [152]. Taken together, S1PR1 is an important regulator of immune cell migration also during cancer progression, while at the same time influencing survival, proliferation and pro-tumor cytokine secretion of tumor infiltrating leukocytes. It is important to note that, besides being involved in the production of tumor-promoting cytokines, S1PR1 signaling was also shown to have anti-inflammatory properties that include the suppression of anti-tumor mediators such as IL-12 and nitric oxide (NO) production by macrophages [54,153,154], as well as the suppression of endothelial cell activation [155]. While these properties of S1PR1 signaling remain to be specifically addressed in tumor models, they indicate that S1PR1-dependent signaling may combine favoring tumor-promoting inflammation with suppressing anti-tumor immunity. According to these observations, a potential of S1PR1 as an immune-oncology drug target by reducing intratumoral accumulation of Tregs and pro-tumor cytokine secretion by macrophages resulting in reduced tumor growth and metastasis is prominently discussed in the literature. Nevertheless, blocking S1PR1 could also prevent infiltration of T cells such as CD8+ cytotoxic T cells into tumors, which are needed to effectively kill tumor cells. Accordingly, further studies are required to address if systemic S1PR1 inhibition or rather cell type-specific blockade of S1PR1 would be beneficial for cancer patients.

#### 4.5.2. S1PR2

Similar to S1PR1, S1PR2 is ubiquitously expressed [156]. In the recent years, studies turned their focus mainly on S1PR2 expressed on tumor cells and cancer-associated stem cells revealing both pro- and anti-tumor functions [157,158]. Consequently, little is known about the role of S1PR2 in tumor-associated inflammation. Since S1PR2 signaling counteracts the pro-migratory functions of S1PR1 in leukocytes, one might argue that antagonizing S1PR2 could serve as potential target in cancer therapy by strengthening the chances to turn immunologically cold tumors into hot tumors. However, the unselective influx of immune cells might also result in the arrival of unwanted cells, including immunosuppressive myeloid cells. Accordingly, subcutaneous injection of lung carcinoma and melanoma cells into *S1PR2*-deficient mice led to accelerated tumor growth, in part due to enhanced infiltration of CD11b+ myeloid cells, which stimulated new vessel formation through secretion of proangiogenic mediators such as vascular endothelial growth factor A (VEGF-A) [159]. Furthermore, pharmacological inhibition of S1PR2, which may increase the attraction of immune cells to the tumor site, may by itself serve as an amplifier for tumor development. This is based on findings that around 25% of human germinal center-derived diffuse large B cell lymphomas show somatic mutations in the *S1PR2* gene, and *S1PR2*-deficient mice develop B cell lymphomas at an advanced age [160]. In summary, further studies are needed to analyze the impact of S1PR2 in immune cell functions in different tumor entities in order to decipher a possible role in turning cold into hot tumors.

#### 4.5.3. S1PR3

S1PR3 also shows a rather broad tissue expression profile similar to S1PR1 and 2. However, S1PR3 appears to be rather selectively expressed in monocytes within human blood peripheral blood mononuclear cells [156]. Similar to S1PR2, as mentioned above, so far most studies addressing the function of S1PR3 in cancer concentrated on its role in tumor cells. There, it was shown that S1P/S1PR3 signaling is a potent driver for tumor cell migration, proliferation, angiogenesis, and metastasis in different tumor entities such as lung and breast cancer [161,162,163,164]. However, little is known about the immune cell specific function of S1PR3. Since studies describing the role of S1PR3 in inflammation point towards a pro-inflammatory function by increasing leukocyte rolling and inducing an inflammatory dendritic cell phenotype that activates CD4+ T cells to produce IFN-γ, one might speculate that S1PR3 activation enhances anti-tumor immunity [165,166]. Indeed, a recent study pointed towards this direction by reporting that S1PR3 signaling activated NF-κB signaling and potentiated the interferon (IFN)-α and -γ response in hematopoietic stem cells (HSC), thereby promoting myeloid differentiation of HSC. Thus, S1PR3 is proposed as both a prognostic marker and novel therapeutic target in acute myeloid leukemia [167]. In summary, whereas S1PR3 activation on tumor cells would most likely worsen cancer prognosis, an immune cell specific S1PR3 agonist might serve as a potential pharmacological drug to enhance anti-tumor immune response. However, further studies are obviously required to test this hypothesis.

#### 4.5.4. S1PR4

In contrast to S1PR1-3, S1PR4 shows a rather specific expression pattern restricted in hematopoietic tissue and endothelium and a lower expression within the gastrointestinal tract, the lung, and the brain [81,168,169]. Whereas the other S1P receptor family members are important for immune cell migration, S1PR4 appears to have a limited function in lymphocyte trafficking in the lymphatics [170]. Rather, S1PR4 plays a role in immune cell activation, particularly in the activation of myeloid cells in a tumor context [104,171]. S1P released by dying MCF-7 breast cancer cells activated S1PR4 on human DCs to release IL-27, which in turn triggered Tregs to suppress CD8+ T cell function, leading to reduced tumor cell killing [172]. Activation of S1PR4 on human plasmacytoid DC (pDC) resulted in diminished CD8+ T cell activation as well, in this case via reduced IFN-α secretion by pDC [173]. Furthermore, S1P-S1PR4 signaling mediated a tumor-promoting phenotype of human and murine macrophages by inducing anti-inflammatory IL-10 production upon translocation of tropomyosin receptor kinase A to the plasma membrane [174]. These in vitro studies suggested a tumor-promoting role for S1PR4 by shifting tumor-associated inflammation towards an anti-inflammatory pattern. Indeed, in murine tumor models of CAC and especially in breast cancer upon standard-of-care chemotherapy, *S1PR4* ablation resulted in significantly reduced tumor growth through enhanced CD8+ T cell proliferation. Unexpectedly, this appeared to be partly independent of myeloid cells since enhanced cytotoxic T cell proliferation was triggered via a T cell-intrinsic mechanism [175]. Importantly, disrupting *S1PR4* on murine T cells also favored memory formation [175]. There is also evidence for a suppressive role of S1PR4 in human T cells, where *S1PR4* was expressed by nutrient-deprived CD8low T cells and increased CXCR4 expression [176]. CXCR4 is a chemokine receptor that is involved in promoting Treg attraction to the tumor site and its blockade enhanced anti-PD-1 therapy in vivo [177,178]. However, more studies are required to clarify the involvement of myeloid cells in S1PR4-dependent establishment of tumor-supporting inflammation. Interestingly, *S1PR4* ablation also enhanced IFN-α/-β production in murine breast tumors, which may aid in increasing intratumoral CD8+ T cell numbers and activity [173,175]. Besides these anti-inflammatory properties, S1PR4 signaling also affected myeloid cell-dependent establishment of TH17 cells, which are well-known drivers of CAC. Lack of TH17 cells may have contributed to reduced tumor growth in the AOM/DSS model of CAC when *S1PR4* was absent [175,179,180]. CD4+ D011.10 T cells produced significantly less IL-17 in co-culture with OVA albumin-pulsed *S1PR4*-deficient DCs, indicating a role for S1PR4 in the induction of TH17 cell differentiation [180]. Due to the above-mentioned studies, which are clearly supporting a pro-tumor role for S1PR4, data correlating *S1PR4* expression with tumor patient prognosis seem rather counter-intuitive. *S1PR4* expression strongly correlated with improved prognosis of triple-negative breast cancer and non-small-cell lung cancer patients, and it was furthermore positively associated with lymphocyte infiltration [136,137]. However, due to the prominent expression of *S1PR4* on lymphocytes compared to other cells, these data likely do not reflect a functional role of S1PR4, but rather indicate that *S1PR4* expression in human tumors can be taken as a marker for tumor-infiltrating lymphocytes. Taken together, these studies underscore the potential of S1PR4 as a possible drug target in immune oncology.

#### 4.5.5. S1PR5

Similar to S1PR4, S1PR5 has a rather restricted expression pattern mostly being expressed by NK cells, T cells, monocytes, and oligodendrocytes [82,181]. The few studies investigating this receptor mainly indicate S1PR5 as a potent driver of NK cell and monocyte trafficking, where the expression of *S1PR5* on NK cells [82,182] and Ly6C- non-classical monocytes was required for their egress from the bone marrow [102]. Interestingly, unlike for NK cells, S1PR5-dependent migration of monocytes seemed to be an S1P-independent mechanism indicating that S1PR5 regulates migration of monocytes and lymphocytes through different mechanisms. Although there is limited data on the function of S1PR5 in tumor-infiltrated leukocytes and its contribution to tumor progression, some recent studies correlated the expression of *S1PR5* on Trm or effector memory T cells (Tem) with cancer prognosis. Single cell RNA sequencing (scRNA-Seq) of T cells from 12 patients with CRC suggested that Tem highly expressed *S1PR5* [183]. Furthermore, MHC class Ib-restricted CD8+ T cells that showed potent anti-tumor efficacy when injected into tumor-bearing mice exhibited high *S1PR5* expression associated with rapid proliferation and prolonged persistence at the tumor site [184]. Thus, *S1PR5* seems to be a potent T cell marker for either effector function or retention as well as T cell homing in tumors. The latter was demonstrated by mass cytometry and scRNA-Seq analysis of hepatitis B virus (HBV)-specific T cells isolated from tumors of HCC patients. There, clusters of HBV-specific Trm T cells were identified that showed reduced expression of *S1PR5* and whose presence correlated with long-term relapse-free survival of cancer patients [185]. In patients with glioblastoma, low expression of *S1PR5* was also associated with improved patient survival, which may fit to observations that glial tumors are enriched in Trm cells [186,187]. In summary, whereas studies on the role of S1PR5 signaling on tumor-specific NK cells are lacking and may be a focus of future efforts, literature indicates that S1PR5 expression dictates the phenotype of tumor-infiltrating T cells with high expression on Tem and low on Trm T cells. The latter is supported by studies showing that *S1PR5* is not only downregulated in Trm, but that its downregulation is a requirement for Trm differentiation [188]. Thus, S1PR5 harbors the potential to be an important marker of tumor-reactive T cells and its inhibition may promote anti-tumor immune memory.

## 5. Conclusions

**Figure 2:**
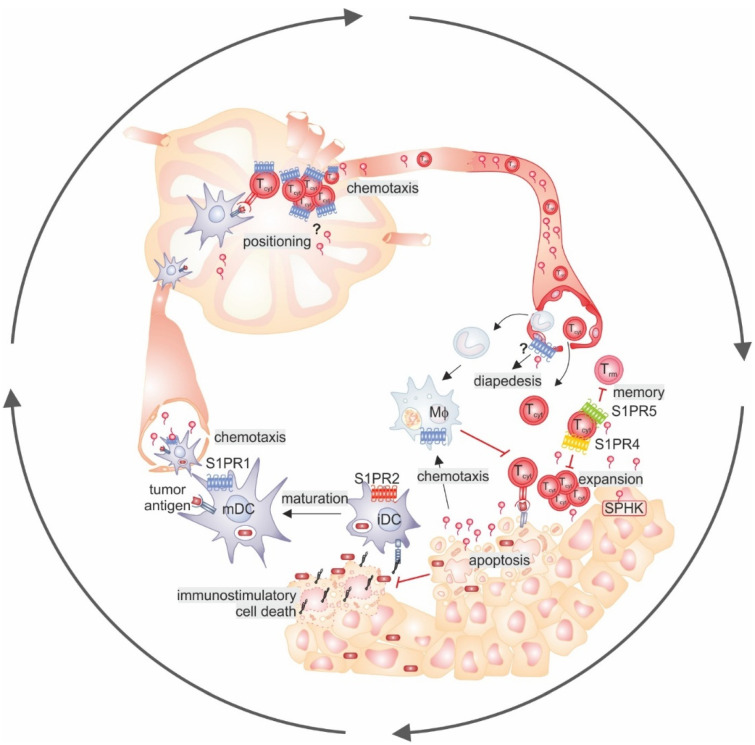
S1P signaling in the cancer-immunity-cycle. Starting from the lower left corner, the maturation of DCs upon antigen uptake in the context of immunostimulatory cell death upregulates S1PR1 to allow DCs to follow the S1P gradient into the lymph. In lymph nodes, local S1P gradients may affect the correct positioning of cells required for cytotoxic T cell activation and expansion. These express S1PR1 to follow the S1P gradient into the blood stream. At the tumor site, stabilization of tumor blood vessels by S1PR1 signaling may affect myeloid versus lymphocyte infiltration (diapedesis). Effector T cell expansion is inhibited by S1PR4, as is memory formation by S1PR5. T cell killing of tumor cells by apoptosis releases S1P which attracts macrophages that can suppress cytotoxic T cell activation. Moreover, they remove apoptotic cells, avoiding the transition to immunostimulatory cell death, thereby hindering DC maturation. Details can be found in the text. Black arrows indicate activation/progression; red arrows indicate inhibition. Abbreviations: DC, dendritic cell; iDC, immature DC; mDC, mature DC; MΦ, macrophage; S1P, sphingosine-1-phosphate; S1PR, S1P receptor; SPHK, sphingosine kinase; Tcyt, cytotoxic T cell; Trm, resident memory T cell.

In our opinion, the data discussed above clearly indicate that targeting components of the S1P signaling machinery may be beneficial not only to alter tumor cell properties, but to create a favorable immune environment. S1P appears to affect virtually all steps of the cancer-immunity cycle [189] (Figure 2).

Tumor cell death by apoptosis and the resulting interaction of apoptotic cells with antigen-presenting cells (APC) promotes immune evasion, tissue remodeling, and an invasive phenotype in tumors [190]. S1P serves as a signal promoting apoptotic cell removal and affecting downstream APC responses [38,191]. Interfering with S1P in this context may result in secondary cell lysis, leading to the release of endogenous danger signals or damage-associated molecular patterns that serve as ligands of immune cell receptors such as toll-like receptors or the receptor of advanced glycosylation end products [3,192,193]. The immune-activating properties of lytic cell death may help to trigger APC activation to induce protective inflammation [6,192]. Once APC are activated, they upregulate S1PR1 to be able to migrate to the lymphatics [105,106,107], where local S1P gradients promote the correct positioning and, thus, interaction of cells to induce potent immune reactions [194]. T cell egress from the lymph nodes again requires S1PR1-dependent chemotaxis. Tumor-associated blood vessel stabilization by endothelial S1PR1 appeared to shift the tumor immune profile from myeloid towards lymphoid cells [118], although it is not clear if that relies on differences in diapedesis across leaky versus stabilized blood vessels. The proper activation of T cells and memory formation at the tumor site are likely limited by signaling through more than one S1PR. This will also depend on immunosuppressive polarization of myeloid immune cells in the TME by S1P [54,195]. Identifying which intervention(s) in the S1P signaling machinery are the most beneficial to keep the cancer-immunity cycle going requires further studying. However, it appears rational to avoid the induction of lymphopenia, as the peripheral immune repertoire predicts patient survival, with lymphopenia being associated with decreased survival [196] both before and after chemotherapy [46]. Importantly, systemic immunity, which requires a functional peripheral immune system, is also imperative for the success of ICB since it allows the development of new immune effectors rather than only reactivating pre-existing cells [48]. These long-term effects are probably underestimated in studies where, e.g., short-term experimental metastasis models are not affected by lymphopenia upon SPNS2 ablation or SGPL1 inhibition [140]. It will be important to determine if increased residency of lymphocytes in these models also confers protection from metastases in more physiologically relevant tumor models. This mechanism may be relevant for keeping tumors in a dormant state, since CD8+ Trm were recently shown to promote immune equilibrium in melanoma [197]. However, increased local levels of S1P were shown to prolong the time T cells spend in lymph nodes, which may improve T cell activation [198]. Irrespective of these remaining questions, the picture emerges that targeting individual sphingosine kinases or selected S1P receptors such as S1PR4 or S1PR5 may have the highest potential in unleashing the power of the anti-tumor immune response. However, aiming at S1P production alone may not be sufficient as indicated by the so far underwhelming results from clinical trials with such compounds. Future clinical trials with compounds affecting circulatory S1P levels should include monitoring their effects on tumor-associated immunity. Moreover, S1PR4/5 selective modulators await identification.

## Data Availability

No new data were created or analyzed in this study. Data sharing is not applicable to this article.
